# Histopathology and cholinergic assessment of *Pterocarya fraxinifolia* on chicken embryo

**DOI:** 10.2478/v10102-009-0023-1

**Published:** 2009-12-28

**Authors:** Parisa Sadighara, Javad Ashrafihelan, Abbas Barin, Tahreh Ali Esfahani

**Affiliations:** 1 Department of toxicology, Faculty of veterinary, Tehran University, Tehran, Iran; 2 Department of pathobiology, Faculty of veterinary, Tabriz University, Tabriz, Iran; 3 Department of clinical pathology, Faculty of veterinary, Tehran University, Tehran, Iran

**Keywords:** *Pterocarya fraxinifolia*, toxicity tests, cholinergic assessment, chicken embryotoxicity

## Abstract

There are no reports of toxicological studies of *Pterocarya fraxinifolia*. The leaves are used for fishing, which also an anesthetic agent. Currently, many drugs utilized in anesthesia practice are modified cholinergic transmission and acetylcholine esterase inhibitors; these are parts of anaesthetic pharmacy. Therefore, cholinergic assessment was surveyed in chicken embryo, which *Pterocarya fraxinifolia* extractes were injected in 0.1, 1 and 10 mg concentration at day 4 of incubation. Serum and brain cholinesterase were analyzed on day 20 of incubation. The signs were not due to the changes of cholinesterase activity. In histopathology examination, massive necrosis was observed in the spinal cord. Other tissues such as heart, kidneys, skeletal bones and muscles, trachea and lungs, digestive system and endocrine glands were completely developed. This data suggests that the spinal cord is a target organ of the bioactive component of this plant.

## Introduction

*Pterocarya fraxinifolia* is a fast-growing tree species naturally distributed throughout Western Blank Sea Region of Turkey and is native to the Caucasus from northern Iran to the Ukraine. Its leaves have antibacterial activity against *Bacillus subtilis*, antifungal activity against *Candida albicans* and *Cladosporium cucumericum*, as well as larvicidal activity against *Aedes aegypti*, and antioxidant (Azadbakht *et al*., 2005). 5-hydroxy-1, 4-naphthoquinone components or juglone that showing antimicrobial activities were found and determined in the leaves of this tree species (Hadjmohammadi and Kamyar, 2006).

The leaves have been used by Caucasian fisherman for catching fish. At present time, native people use these leaves as an anesthetic agent for fishing and for dyeing. Clinical observations showed this compound is remarkably similar to anesthetic compounds.

Many drugs currently used in anesthesia practice modify cholinergic transmission. Acetylcholine esterase inhibitors are part of the anaesthetic pharmacy (Kleinschmidt *et al*., 2005). AChE is an enzyme involved in cholinergic and non-cholinergic functions in both the central and peripheral nervous system (Inestrosa *et al*., 2005). Therefore, our major hypothesis in the pathogenesis of neurotoxicity of this plant was anticholinesterase activity.

The purpose of this study was to evaluate neurotoxicity potential of this plant based on cholinesterase activity and histopathology.

## Materials and methods

### Plant material

The plant was collected in North of Iran and identified by Dr.Gholamreza Amin. Voucher specimen of this plant was deposited in the central herbarium of medicinal plants, Tehran University of Medical Sceiences. *Pterocarya fraxinifolia* leaves were separated, air dried in the shade, powdered and extracted in a soxhlet apparatus with a mixture of methanol-acetone-water (1:1:2 v/v/v) for 24h. The polar and non-polar compounds will be extracted by combination of different of solvents. After that time, the solvents are removed on a rotary evaporator.

### Toxicity tests

#### Chicken embryotoxicity study

Protocol of Chicken embryotoxicity study has gained acceptance by several regulating agencies (Anonymous, 1996). Fertile leghorn eggs were obtained from a breeding farm (Iran farm) on the second day. The eggs were injected on the fourth day. Extracts of *Pterocarya fraxinifolia* was injected in 0.1, 1 and 10 mg concentration into the egg yolk. The dead embryos were fixed in 10% neutral formalin for microscopic examination. The experiment was terminated on day 20 of incubation. Embryos were removed; decapitated, observed malformation and gross lesions. Blood and brain of living embryos were collected for biochemical analysis.

### Cholinergic stress

#### Serum and brain cholinesterase assay

The measurement of cholinesterase is well established. Potassium-phosphate buffer and DTNB was added to samples. Acetylthiocholine iodide was then added and the absorbance at 412 nm was read by spectrophotometer (Ellman *et al*., 1961).

### Microscopic lesion

The tissue samples of dead embryos were fixed in 10% neutral formalin, embedded in paraffin, sectioned at 6 microns and stained with H&E for histopathology.

## Results

Results of Cholinergic Stress are presented in Table 1. Administration of the highest dose of the extraction resulted in 100% mortality of the embryos. In the treatment groups with concentrations of 0.1mg and 1 mg, rate of mortality was 50%. However, high mortality will be observed if embryos are exposed to high concentration of a xenobiotic.

**Table 1:** Effects of *Pterocarya fraxinifolia* on chicken embryos.

	Control	0.1 mg/egg	1 mg/egg	10 mg/egg
N	10	10	10	10
Survival (%)	90%	50%	50%	0
Serum cholinesterase assay (IU)	0.34±0.08	0.41±0.17	0.42±.012	0
Brain cholinesterase assay (IU)	0.68±0.07	0.57±0.1	0.71±0.09	0

There was no significant difference (*p*<0.05) in serum and brain cholinesterase activity. Brain cholinesterase activity was also not in a dose-response depended fashion between groups.

**Figure 1:**
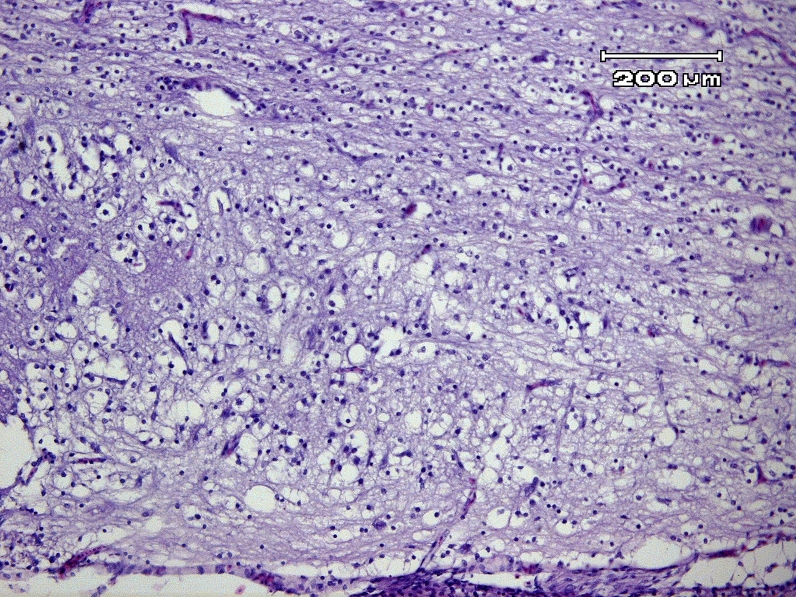
Embryo of fertile Leghorn egg exposed to Pterocarya fraxinifolia extract. White matter of the spinal cord shows primary malacia and vacuolization (leukomyelomalacia). Large, irregular and empty cavities are affected axons and presumably the result of pooling of myelin and liquefaction necrosis. Spongy appearance is presented (lower part of the figure), in contrast, relatively normal white matter in which myelin sheaths are uniform in diameter (upper part of the figure) (H&E,×100, Bar = 200 µm).

The microscopic section is showed in Figure 1. Primary malacia areas in the spinal cord as several ragged cavities scattered through various white columns especially in the dorsal funiculli of cervical region but were not symmetrical. In these foci of malacia, the large, irregular, empty cavities were presumably the result of pooling of myelin and liquefaction necrosis. The necrosis in spinal cord was considerable. Swollen axons were detected in cross section. These affected axons and were appeared as spherical, darkly stained structures or were absent. In sections cut of parallel to the length of the tract, axons were distorted, thickened in some segments and were fragmented at other points along the course and this tissue was vacuolated, spongy appearance, in contrast, normal white column in which myelin sheaths were uniform in diameter.

There was no decrease in hematopoiesis (granulopoiesis and erythropoiesis) in the bone marrow and extramedullary hematopoiesis centers within other tissues. Other tissues such as, heart, kidneys, skeletal bones and muscles, trachea and lungs, digestive system and endocrine glands were completely developed.

## Discussion

In this study, for the first time, we carried out toxicology study of *Pterocarya fraxinifolia* with regard to cholinesterase activity and histopathology. It was supposed that this plant might inactivate cholinesterases. The hypothesis that this plant might inactivate cholinesterase was supported by studies that cholinesterases are measured as clinical parameter in neurotoxicity of anesthesia drugs. The studies had shown that barbiturate inhibits the specific activity of acetyl cholinesterase. Erythrocyte cholinesterase (E-ChE) activity was markedly reduced (20–40%) after anaesthesia with CO_2_ (Deckardt *et al*., 2007). Some central depressants (e.g. chlorpromazine) reduced the appearance of acetylcholinesterase in the cerebrospinal fluid (Vogt *et al*., 1984). Nevertheless, in present study, we illustrated that the changes of acetylcholine esterase activity were not significant.

Morphological examinations are important in establishing the precise site of toxic lesions (Lu, 1996). Histopathology helps in identifying target organs of toxicity and mechanism of action (Wester *et al*., 2002). In histopathology examinations considerable liquefaction necrosis of the spinal cord and myelinated degeneration area were observed. There were no special histopatological changes in other organs.

Up to date, no toxicity tests have been performed on *Pterocarya fraxinifolia*. The results obtained in this study did not confirm neurotoxicity on chick embryo via cholinesterase activity and the observed signs were not due to the changes of cholinesterase activity. But, the spinal cord was microscopically identified as target organ.

Certainly, other *in vitro* and *in vivo* toxicological studies of *Pterocarya fraxinifolia* are needed. These assessments will provide the pharmacological safety dose, toxicity dose, and efficacy data from this plant. In the last decades, plant research has revealed several chemical compounds with important pharmacological activities. Some have been incorporated into drugs (Varanda *et al*., 2006). Therefore, it is possible that potential components with pharmacological effects could be isolated from *Pterocarya fraxinifolia*.
